# Chemical Vapor Deposition Synthesis of 3D Hybrid Carbon Materials at Low Pressure and Temperature

**DOI:** 10.3390/molecules31162826

**Published:** 2026-08-13

**Authors:** Carolina Rojas, Neida Santacruz, Frank Mendoza, Sebastián A. Michea, Gerardo Morell, Brad R. Weiner

**Affiliations:** 1 Department of Chemistry, College of Natural Sciences, University of Puerto Rico, Rio Piedras Campus, San Juan, PR 00925-2537, USA; brad.weiner@upr.edu; 2 Department of Physical Sciences, College of General Studies, University of Puerto Rico, Rio Piedras Campus, San Juan, PR 00925-2532, USA; neidamirey@gmail.com; 3 Department of Physics, College of Natural Sciences, University of Puerto Rico, Mayagüez Campus, Mayagüez, PR 00681-9000, USA; frankmendoza1@gmail.com; 4 Instituto de Ciencias Aplicadas, Facultad de Ingeniería, Universidad Autónoma de Chile, Santiago 8580658, Chile; sebastian.michea@uautonoma.cl; 5 Department of Physics, College of Natural Sciences, University of Puerto Rico, Rio Piedras Campus, San Juan, PR 00925-2537, USA; gerardo.morell@upr.edu

**Keywords:** carbon materials, hybrid carbon materials (HCMs), CVD, scaffold, novel synthesis method, low pressure

## Abstract

A novel low-temperature method to synthesize a hybrid carbon material (HCM) is reported. The synthesis relies on a chemical vapor deposition (CVD) approach, using zeolite NaY as a scaffold structure and acetone as the carbon source. The deposition temperature was 500 °C at a pressure of 46 ± 1 kPa. Following the growth of the HCM, the material was treated with HF to remove the scaffold, and the resultant materials were characterized by high-resolution scanning electron microscopy (HR-SEM), energy-dispersive X-ray spectroscopy (EDS) mapping, transmission electron microscopy (TEM), X-ray diffraction (XRD), Raman microscopy, and Brunauer–Emmett–Teller (BET) surface area analysis, showing heterogeneous morphology. The results demonstrate that synthesis at low temperatures produces a robust hybrid material that can adopt different configurations and/or morphologies by modifying the scaffold structure.

## 1. Introduction

As one of the most abundant elements in nature, carbon exists in several different allotropes, such as diamond, graphite, fullerenes, and graphene [1], with different physical and chemical characteristics. The groundbreaking experiments by Novoselov and Geim [2], in 2004, led to the discovery of graphene as a monocrystalline graphitic film, which in turn led to new functionalized forms, e.g., graphene oxide and reduced graphene oxide [3]. To date, this broad spectrum of carbon nanomaterials is the focus of intense research interest due to their practical applications in semiconductors, batteries, sensors [4], environmental remediation [5], and biomedicine [6]. Significant interest in carbon nanomaterials is attributed to their unique properties for [7] electrodes [8], conductors, semiconductors, and in optoelectronic applications [9].

The synthesis of carbon nanomaterials follows two principal approaches: exfoliation methods and growth/molecular assembly processes. The exfoliation methods involve separating a single layer of carbon material either mechanically (e.g., using adhesive tape to peel graphene from graphite) [10] or chemically (e.g., the Hummers method for graphene production) [11]. The growth/molecular assembly methods include laser ablation, arc discharge, chemical vapor deposition (CVD) [12], and electrospinning [13]. In this work, we have used CVD as our growth method of choice.

Metallic catalysts, e.g., nickel [14], cobalt [15], iron, platinum, palladium, and gold [16], are often employed in the growth/molecular assembly methods. The iron group (Fe, Co, Ni) and their alloys have been the most useful catalysts in the synthesis of carbon nanotubes [17], while graphene is typically obtained using copper and nickel [18]. Due to the superior solubility and diffusivity of carbon on nickel and the inherent catalyst capacities of Ni, the metal can be combined with porous materials such as zeolites or oxides [19] to grow carbon allotropes. The carbon source adsorbs and dissociates on the nickel metal, which diffuses along the surface until saturation is reached. At this point, the carbon can precipitate from the metal and form nanoparticles. This process can depend on several parameters, including the physical nature of the catalyst, the synthesis temperature, and the precursor gas [20].

While many of these nanoscale materials have practical applications in their “pure” form, different growth methods, such as CVD combined with porous materials, offer the possibility of growing hybrid carbon nanomaterials, i.e., composed of different morphologies [21]. These morphologies can include graphene, graphene oxides, carbon nanotubes, and carbon nanofibers, among others. As a result, the hybrid carbon material (HCM) has demonstrated a wide range of applications, e.g., textiles [22], separations [23], and environmental remediation [24]. A mix of graphene-like materials, graphene oxide-like materials, and carbon nanotubes provide the hybrid character [25].

Adjusting experimental conditions enables the formation of a mixture of these morphologies within porous materials, resulting in a three-dimensional structure [26]. Whereas the carbon source may exist in any physical state, the feedstock employed in these types of synthesis is commonly gaseous, e.g., methane (CH_4_) [27], ethylene (C_2_H_4_) [28], and acetylene (C_2_H_2_) [29]. Solids are in general used as dopants in bulk metals [30], e.g., amorphous carbon [31] and camphor [32]. Also, liquids including ethanol [33], isopropanol, and acetone [34] have been used, but in smaller amounts. One of the interesting industrial processes is acetone pyrolysis. This reaction has been extensively studied to understand its kinetics, mechanisms, and side products [34]. Acetone pyrolysis can be carried out between 500 and 700 °C; at these temperatures acetone is the gas phase (vapor pressure = 231 mm Hg @ 25 °C) [35] which ensures that it is available for the pyrolysis reaction. Thus, in practical terms, acetone is available, inexpensive, and there is no need for restriction or special permission [36].

Chi and Kim [37] found that the synthesis of carbon nanomaterials using zeolite frameworks depends on the chemical properties of the porous framework, including the Si/Al ratio, the cation type, heat stability, the carbon introduction method, and the porosity development method. They concluded that the optimal zeolite type features a pore-entrance formed by Si-O-Si rings, ideally with three-dimensional pore networks and oxygen in a 12-membered ring. Examples that fit this description include BEA (zeolite Beta) [38], EMT (or EMC-2 (two), École Supérieure Mulhouse Chimie) [38], and FAU (Faujasite) [39]. For the Si/Al ratio, the recommendation is to use a value of 20 or less to obtain the desired results. Considering that the zeolite Al site carries a negative charge, a cation is needed to balance this charge [36,40]. To obtain high-quality HCMs according to Chi and Kim [37], the observed pyrolysis temperatures must be >800 °C and under 900 °C. Nishihara et al. reported that these synthesis techniques produce three types of carbon materials [40]. Type I forms a three-dimensional organized framework by directly replicating the zeolite structure [41]. In type II, carbonaceous components mixed with type I and non-organized carbon templates, such as zeolite, are observed [41]. Finally, type III rarely exhibits as type I and is observed as a disordered structure. X-ray diffraction (XRD) patterns and surface area (BET) measurements are the techniques that allow identification of the type of carbon materials obtained [36,41]. Overall, type I presents an organized and ordered structure, with the carbon XRD (002) peak absent, and a surface area >2100 m^2^·g^−1^; type II shows an organized structure copied from zeolite and some graphene structures that include the XRD carbon (002) peak, and has a low surface area <2100 m^2^·g^−1^; and type III presents disorder structures, and may contain the XRD (002) peak of carbon [36,42].

The synthesis of HCMs via CVD Is governed by several physicochemical parameters that control the morphology, composition, and quality of the material. Among these, the synthesis temperature influences carbon diffusivity and may affect the catalyst activity [43]. Depending on the carbon precursors and the framework, this temperature can vary between 500 and 1200 °C [44]. System pressures play an important role in optimizing carbon diffusion within the zeolite scaffold while minimizing undesired side reactions. Lower pressures facilitate the scaffold replication and order the material formation [45]. The zeolite framework properties, i.e., pore size, Si/Al ratio, and cation type, directly affect the carbon distribution and determine the resulting carbon typology [42]. Finally, the identity of the carbon precursor, its volatility, and reaction time govern carbon solubility in the catalyst metal surface and the selectivity toward the specific carbon allotropes formation (i.e., graphene, CNTs, CNFs) [34]. The analytical selection of these parameters will optimize the expected HCM, and in turn influence practical applications. In this paper, we present the synthesis of a hybrid nanomaterial by chemical vapor deposition using acetone as a carbon source under low pressure and temperature [36]. As a result, we obtain a type II material. The carbon composition includes graphene, graphene oxide, carbon nanofibers (CNFs), and carbon nanotubes (CNTs). The key innovation here is that the resulting material is obtained at substantially lower temperatures and pressures than have previously been reported, which may have important implications for scaling up and future industrial production [36]. The resulting material has many potential applications, e.g., antimicrobial activity [42], plant growth [46], energy storage [47], gas adsorption [48], and water purification [49], to name a few.

## 2. Results and Discussion

The obtained material is a black powder. The synthesis reaction yield was computed by comparing the actual carbon yield and the theoretical carbon yield (see (1)). By considering the inlet volume of the acetone vapor and the mass of powder obtained, the synthesis reaction yield (*SRY*) was 35.12 ± 5.17%, assuming the product contains 85.26% carbon (value obtained from EDS scanning) [36]. Previous studies have reported SRY in the 10–70% range; however, they omit the working pressure at which it was obtained [50].

(1) $$\mathrm{SRY}=\frac{\mathrm{Actual}\;\mathrm{yield}}{\mathrm{Theorethical}\;\mathrm{yield}}\times 100$$

### 2.1. High-Resolution Scanning Electron Microscopy (HR-SEM)

**Figure 1:**
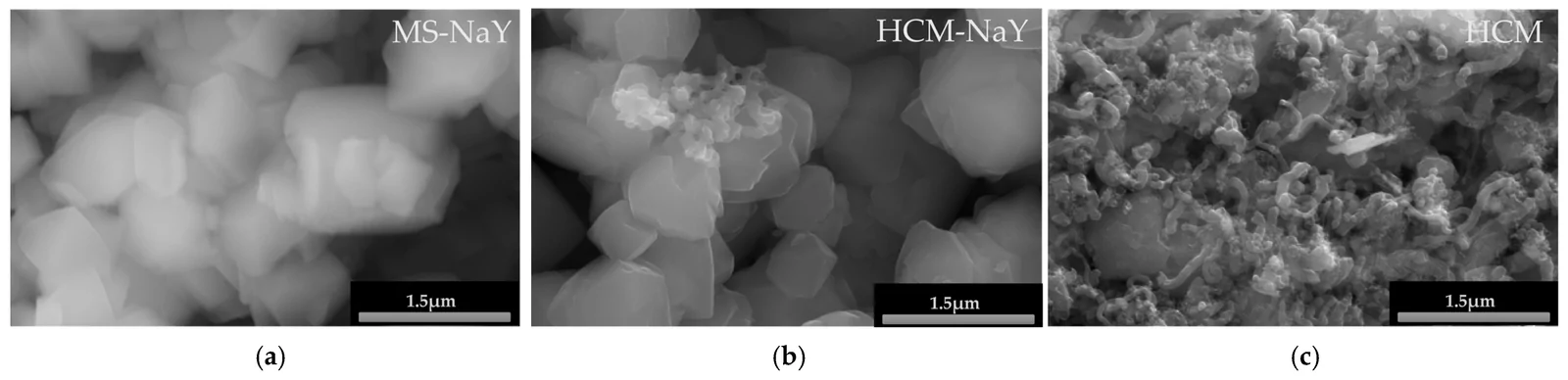
HR-SEM images of (**a**) zeolite NaY only; (**b**) the product of the synthesis, zeolite plus carbon; and (**c**) the hybrid carbon material (HCM) without zeolite. The scale is 1.5 µm, and the magnification is 100,000×.

The high-resolution scanning electron microscopy photographs were obtained at 100,000× magnification and an acceleration voltage of 20 kV. Figure 1 exhibits the morphological differences between zeolite-NaY, zeolite-NaY with carbon material, and hybrid carbon material only. Figure 1a presents the structural faces of zeolite and different framework geometries prior to synthesis. Figure 1b exhibits a “spaghetti”-like morphology obtained from the framework and the integrated carbon material, confirming that the synthesis occurs. In addition, the working temperature (500 °C) of our system [36] supports the growth of carbon nanofibers (CNFs) [51]. Carbon nanofibers exhibit stacked cone or cup structures with graphene layers at angles of 4–36° with respect to the fiber axis and diameters in the range of 70–200 nm [52]. Carbon nanotubes (CNTs) are symmetric cylinders composed of graphene shells rolled along their own axis (cone angle = 0°). The diameters of the single-walled carbon nanotubes (SWCNTs) are between 0.4 and 3 nm, and multiwalled carbon nanotubes (MWCNTs) are typically between 2 and 200 nm [53]. These differences allow us to differentiate between CNTs and CNFs. Figure 1c exhibits the HCM free of zeolite following the hydrofluoric acid treatment. Here, CNTs and CNFs with different diameters, resulting from the zeolite channel geometries, are shown. Based on our observations of the CNTs and CNFs, we propose that the carbon material enters the framework channels, fills them, and then covers the rest of the framework. After the HF treatment removes the zeolite scaffold, the carbon structure remains intact, with no apparent damage. The shape adopted by the carbon structure (see Figure 1c) indicates that zeolite NaY serves as an appropriate scaffold for nanomaterial synthesis [36].

### 2.2. Energy-Dispersive X-Ray Spectroscopy

Two X-ray spectroscopic tools were used to characterize the material: SEM-EDS analysis and EDS mapping. SEM-EDS was used to perform qualitative and quantitative elemental analyses of the sample at a micrometric spatial resolution. EDS delivers an elemental composition of the sample. Mapping scans the sample’s surface and detects the location of each element, providing a spatial distribution of the element [54]. This analysis allows us to understand the hetero- and/or homogeneity of the sample.

**Figure 2:**
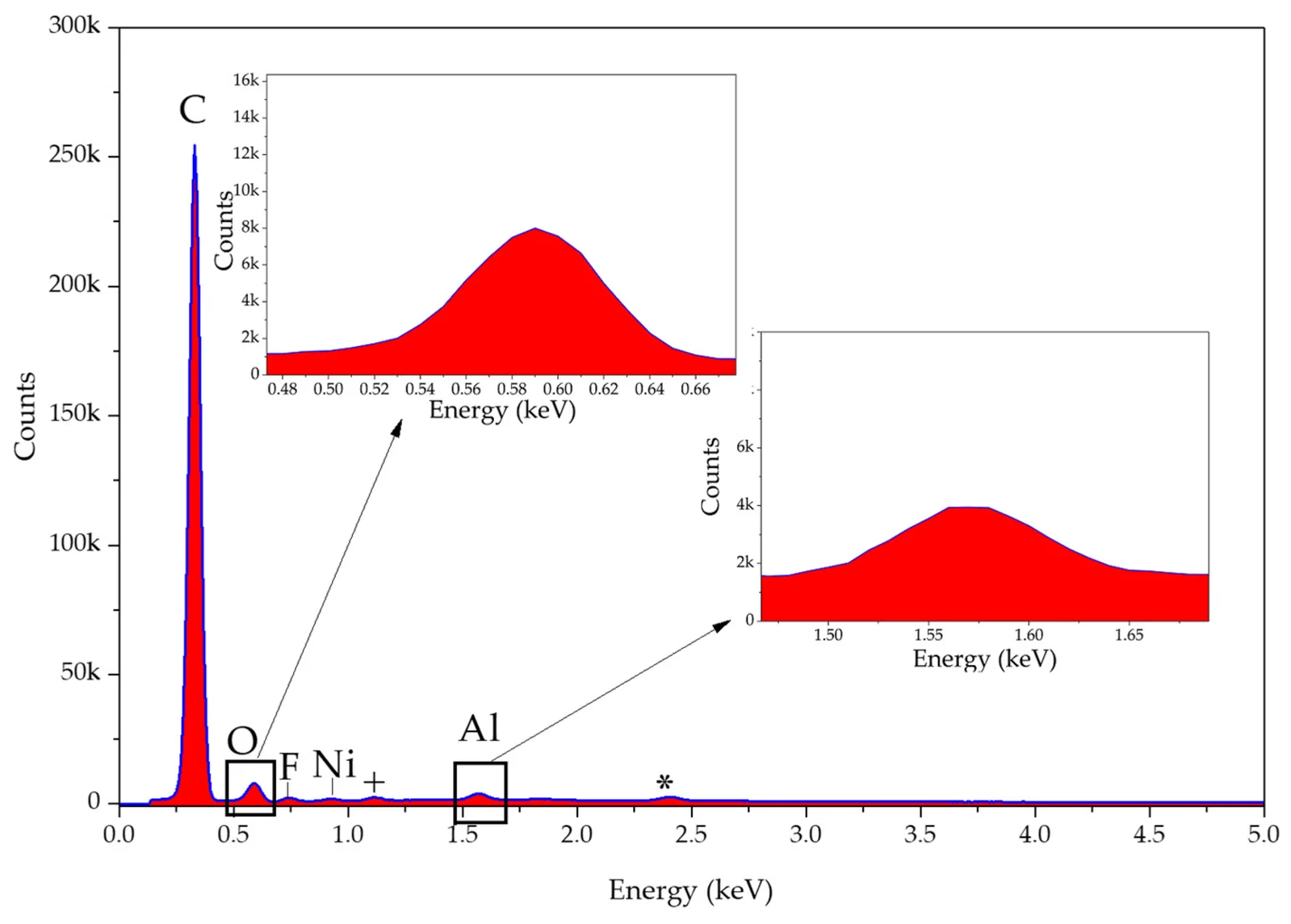
EDS plot corresponding to the HCM. The inset plots correspond to the expansion of the oxygen and aluminum regions of the spectrum. The peak marked with a + most likely corresponds to a residual sodium signal (Na Kα 1.041 keV and Kβ 1.071 keV) [55], from the zeolite scaffold. The peak marked with an * most likely corresponds to contamination by sulfur (Kα 2.307 keV and Kβ 2.464 keV) [56].

EDS analysis was performed at 5000× magnification with 20 kV of acceleration voltage, at a resolution of 960 × 600 pixels (107.9 nm/pixel), with a dwell time of 200 ns (per pixel per frame), for an acquisition time of 45 s. The EDS results are shown in Table 1 and Figure 2. Table 1 shows the composition of the samples before and after HCM synthesis, and of the HCM after HF treatment. Besides the elements included in Table 1, the EDS analysis detected trace amounts of nickel (0.68 ± 0.01%) and fluorine (1.59 ± 0.01%) (see Figure 2).

**Figure 3:**
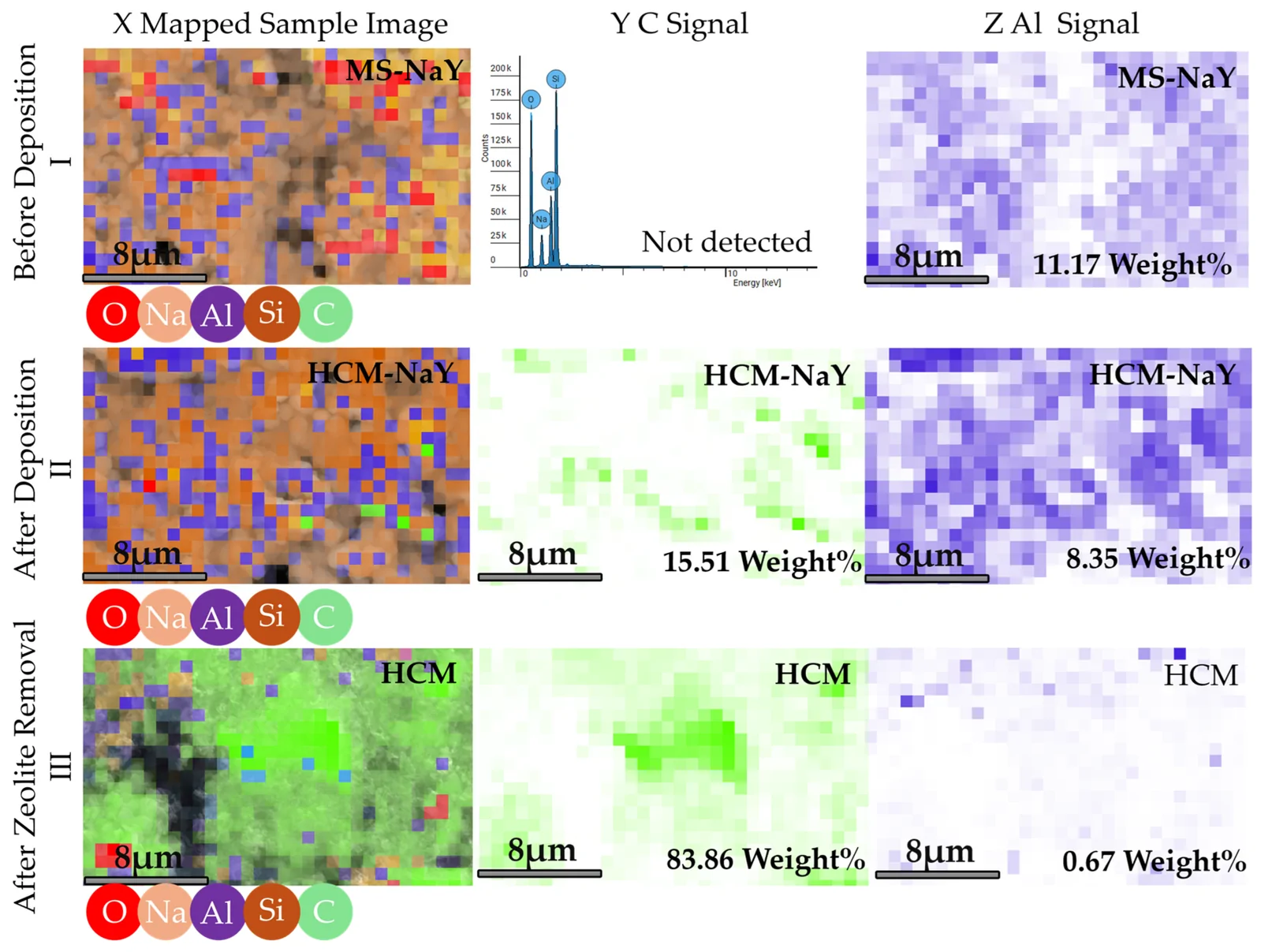
EDS mapping shows, in the first column from the top down, zeolite only (MS-NaY) (XI), zeolite–carbon material (HCM-NaY) (XII), and pure carbon material (HCM) (XIII) mapping images. The second and third columns correspond to the mapping of carbon (Y column) and aluminum (Z column), respectively. The colors in the scanned images correspond to red: oxygen, orange: sodium, violet: aluminum, brown: silicon, green: carbon. Row I shows the zeolite scan, row II corresponds to the material with zeolite (HCM-NaY), and row III corresponds to the zeolite-free material (HCM). The scale of the images is 8 μm, the magnification is 20,000×, and the resolution is 960 × 600 pixels (26.98 nm/pixel), with a dwell time of 200 ns (per pixel per frame), for an acquisition time of 64 s.

The EDS mapping was performed at 20,000× magnification and 20 kV of acceleration voltage, at a resolution of 960 × 600 pixels (26.98 nm/pixel), with a dwell time of 200 ns (per pixel per frame), for an acquisition time of 64 s. The resulting data are shown in the matrix of Figure 3. The mapping illustrates the spatial distribution of the elements across the material surface. The X column corresponds to the mapped SEM images. Starting with zeolite NaY (Figure 3, XI), the carbon signal (Figure 3, YI) is not detected. After the synthesis (row II), the carbon content of the material corresponds to 15.51 ± 0.02% by weight.

Following treatment with hydrofluoric acid, i.e., removal of the inorganic scaffold, the carbon content increases to 83.86 ± 0.43%. Figure 3 (ZIII) shows the presence of aluminum in the sample. Aluminum is chosen to monitor zeolite behavior for practical reasons, but choosing either elemental Na or Si would give similar results [36]. After the HF treatment (HCM sample), mapping detected a small amount of fluorine (2.10 ± 0.43%), attributed to the treatment used to remove zeolite. Furthermore, mapping also detected traces of Al (0.67 ± 0.05%) and Na (0.67 ± 0.05%), corresponding to a zeolite remnant that the acid treatment failed to remove. In addition, the EDS mapping detected a trace amount of Ni (0.60 ± 0.14%). Metallic nickel serves as support for zeolite and most likely acts as a catalyst in the formation of the carbon hybrid materials [57].

The trace amounts of fluorine in the structure most likely do not interfere due to its low concentration and lack of observed interference in both our XRD and Raman spectroscopy results.

### 2.3. Transmission Electron Microscopy (TEM)

**Figure 4:**
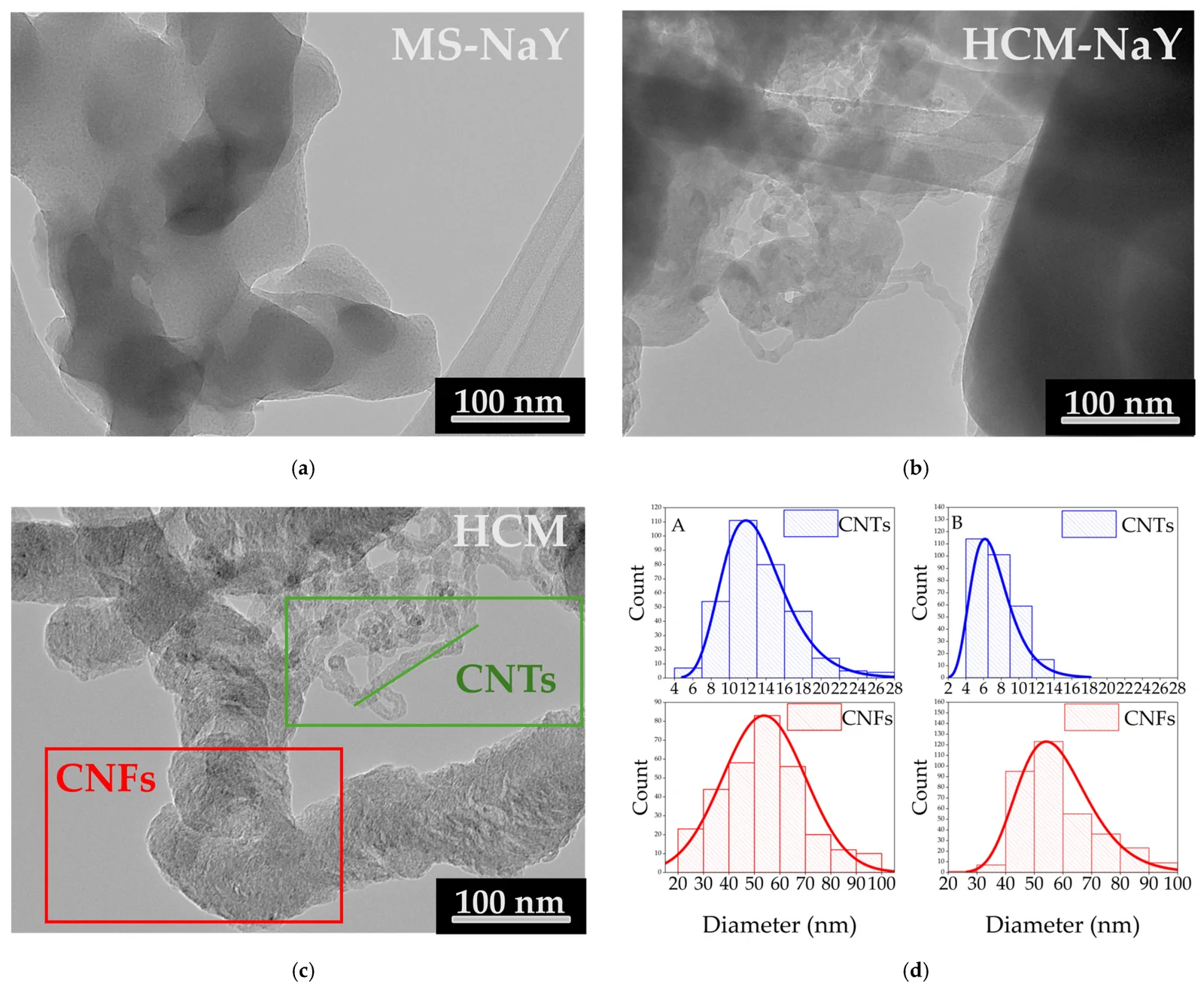
TEM images for (**a**) zeolite NaY, (**b**) zeolite–carbon material, (**c**) hybrid carbon material, and (**d**) histogram distribution plots. In (**c**), CNTs appear hollow and have a smaller diameter in the green inset box; in contrast, CNFs have a larger diameter and appear more robust in the red inset box. Plots (**A**,**B**) within the (**d**) box correspond to the size distribution of the carbon nanotubes and carbon nanofibers before and after the treatment with HF, respectively [36].

The transmission electron microscopy images for zeolite NaY, zeolite NaY with carbon material, and free HCM are presented in Figure 4. The images are consistent with those observed in Figure 1 (HR-SEM images) [36]. We observe the growth of two types of structures, carbon nanofibers (CNFs; red inset, Figure 4c) and carbon nanotubes (CNTs; green inset, Figure 4c). To differentiate between carbon nanotubes and carbon nanofibers, one must observe the angle of inclination with respect to the axis. For example, CNTs coil coaxially, forming a hollow channel, resulting in a concentrically aligned cylinder. CNFs coil at an angle with respect to the fiber axis. This coiling type results in herringbone, platelet, or stacked-up fibers, with no hollow channel observed [58]. HR-TEM and Raman spectroscopy are the most efficient methods for determining structural differences [36,59].

Observing the HCM-NaY sample (Figure 4b), a size distribution with an external average diameter between 13 nm for CNTs and 53 nm for CNFs is observed (see Table 2). In the case of HCM, the external average diameters are 7 ± 2 nm (CNTs) and 58 ± 12 nm (CNFs).

Ziebro and co-workers [60] produced multiwall carbon nanotubes (MWCNTs) using ZSM-5 as a framework, a nickel salt as a catalyst (nitrate), and methane as feedstock in a two-step synthesis method (impregnation followed by chemical vapor deposition at 600 °C). Based on this evidence, our synthesis most likely produces carbon nanotubes and carbon nanofibers with diameters between 4 and 26 nm for CNTs and between 36 and 99 nm for CNFs. The distribution of the diameters is shown in Figure 4d. The carbon nanofibers exhibit a typical herringbone structure [60], in which the graphitic layers are oriented at an angle to the fiber axis [61].

To evaluate the statistical distribution of CNT and CNF sizes, 300 diameters were measured manually using ImageJ 1.52a (https://imagej.net/ij/). Sixty diameters were chosen from five different pictures for each histogram. The histograms are shown in Figure 4d.

### 2.4. X-Ray Diffraction

**Figure 5:**
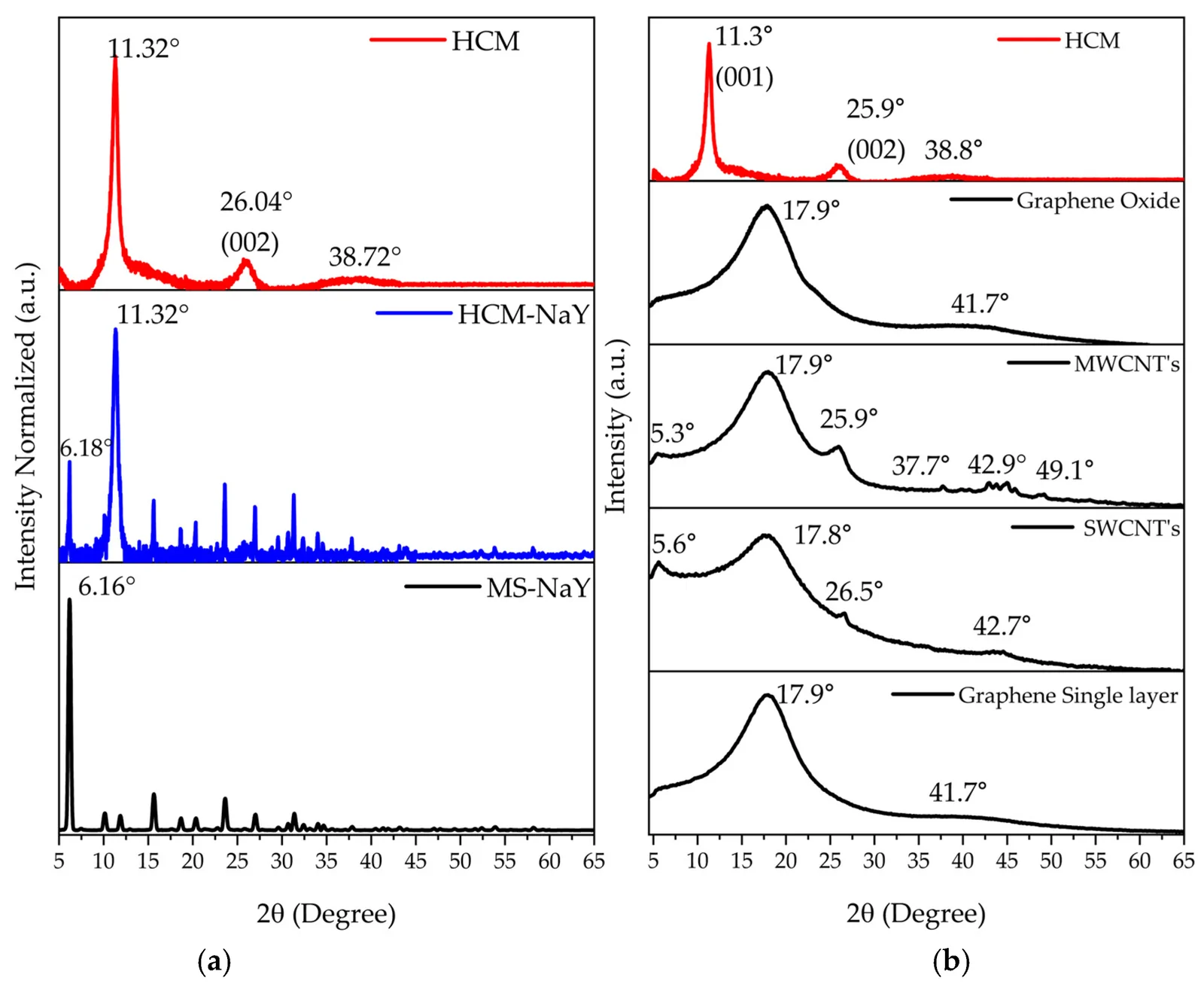
Graph of XRD. Plot (**a**) corresponds to the signal of zeolite NaY (black line), zeolite with carbon (blue line), and hybrid carbon material (red line). Plot (**b**) corresponds to XRD patterns, which compare carbon material (red line) with commercial carbon allotropes (black lines).

Figure 5 displays the X-ray diffraction results obtained in this study. The analysis was measured from 5° to 65° for 2θ. The images show diffractograms of zeolite NaY, HCM-NaY, and HCM. Figure 5a presents pure zeolite NaY in black. The blue line represents zeolite with the carbon material, and the red line represents the free HCM [36].

According to results reported by Johra and co-workers [62], the signal peak at 2θ = 11.32° is distinctive of graphene oxide. The signal at 2θ = 26.04° (002) is indicative of the presence of graphene [62]. The characteristic (002) graphite peak is detected at low intensity and is visible due to the presence of some graphene sheets in the material. According to the literature, the peak displacement observed between 2θ = 11–17° reflects a reduction in interplanar spacing due to the uptake of water molecules by the material [62]. The graph in Figure 5b compares single-layer graphene, SWCNTs, MWCNTs, graphene oxide, and a free hybrid carbon material (red line) [36]. Graphene is a unique graphite film; the name graphene can be used for up to 10 sheets, after which it is called graphite [63].

To obtain the crystallite size, the Scherrer equation was employed (see (2)). *λ* corresponds to the wavelength used in the diffractometer, *β* represents the value of FWHM (in radians) at maximum intensity, and *θ* is the angle of the maximum intensity (in radians). The results are summarized in Table 3.

(2) $$D=\frac{0.9\lambda }{\beta \cos\theta }$$

The results allow for obtaining an approximation of the degree of oxidation in the samples. With an increase in d-spacing and crystallite size, it is possible to see disorders/defects caused by oxygen in functional groups (C-Ox) which intercalate into the carbon structure [64]. It is worth noting that the Scherrer equation brings information related to the crystalline size in a sample powder mix and not necessarily about the particle size [36].

The interplanar distance of GO and graphene was calculated using the Bragg law (see (3)), where the diffraction index (*n*), the X-ray wavelength (*λ*), the peak position (*θ*) and the interplanar distance (*d*) are employed. Table 3 presents our results alongside those reported by Kaushal and co-workers [65]. Consistent with findings by Guerrero-Contreras and co-workers [66], the larger d-spacing observed in GO is attributed to the incorporation of oxygen and associated functional groups [36].

(3) nλ=2dsinθ

**Table 3:** Values of interplanar distance and crystallite size obtained with Scherrer and Bragg equations from the hybrid carbon material.

Sample	Peak 2θ (Degree)		d-Spacing (Å)		Crystallite Size (nm)
	This Study	Other Studies	This Study	Other Studies	
GO	11.32 [36]	10.23 [66]	7.80 [36]	8.64 [65]	8.06 [36]
Graphene	26.04 [36]	26.34 [66]	3.41 [36]	3.38 [65]	5.82 [36]

### 2.5. Raman Spectroscopy

**Figure 6:**
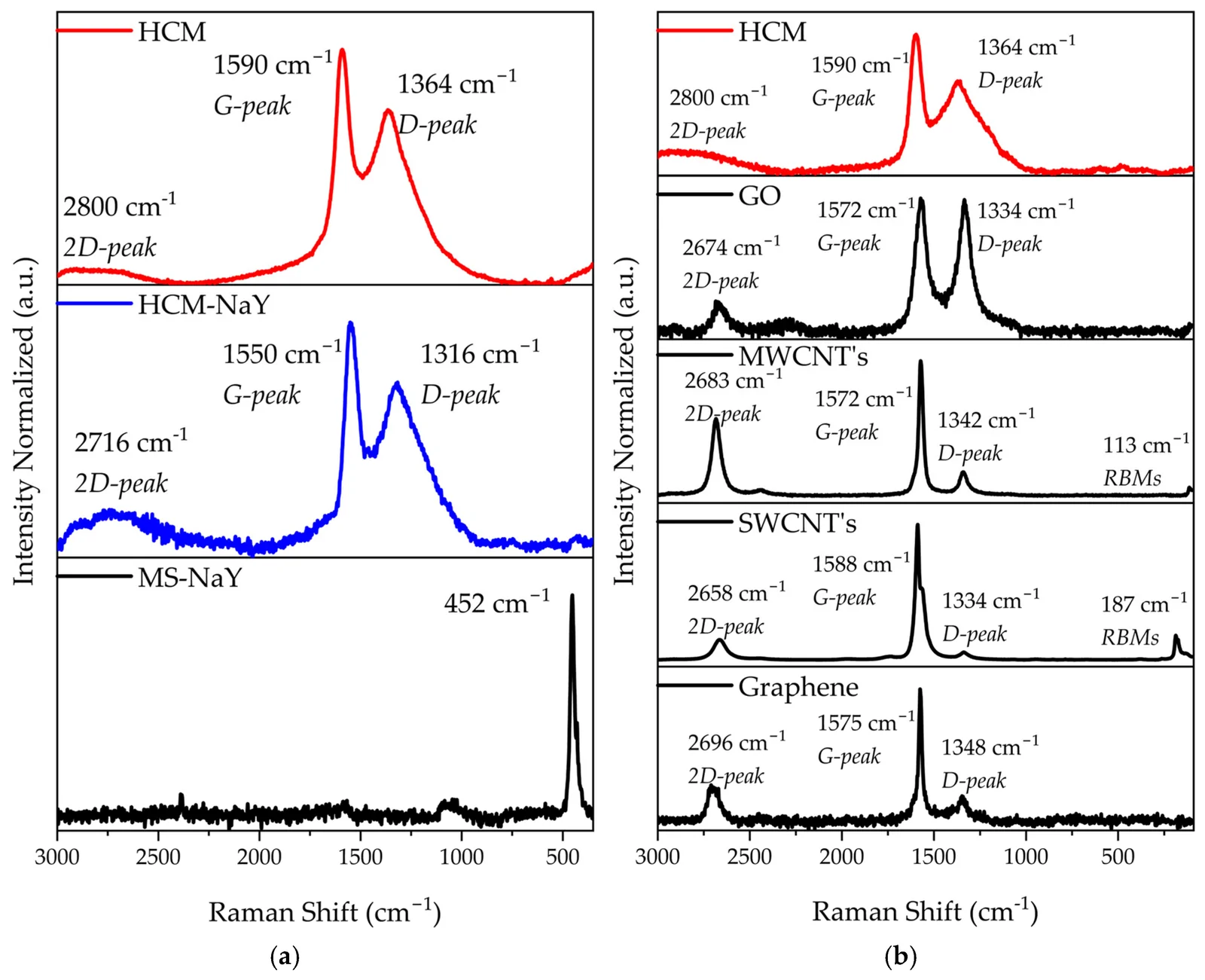
Graph of Raman microscopy analysis. (**a**) corresponds to zeolite NaY (black line), HCM with zeolite (HCM-NaY) (blue line), and free hybrid carbon material (HCM) (red line). (**b**) compares HCM with different commercial carbon materials. RBM corresponds to the Radial Breathing Mode of carbon nanotubes.

Raman spectroscopy was carried out using a 532 nm excitation laser at 10 mW. Figure 6a shows the zeolite NaY (black), hybrid carbon material with zeolite (blue), and the free hybrid carbon material (red) [36]. The red line shows the D-, G-, and 2D-peaks, which are the primary spectral characteristics of graphene. This G-peak originates from the in-plane vibration of sp^2^-bonded carbon atoms [65], and is observed near 1590–1600 cm^−1^. The D-peak is around 1340–1370 cm^−1^ and corresponds to structural defects arising from bonding with hydroxyl and epoxide groups. A comparison of the red (HCM) and blue (HCM-NaY) line Raman spectra shows a shift in the D (~48 cm^−1^) and G (~40 cm^−1^) to higher frequencies upon zeolite removal, indicating a structural relaxation after the HF treatment, confirming that the carbon material covers or wraps the zeolite surface [67]. In addition, the shift in the 2D band position (~84 cm^−1^) to higher frequencies reflects primary structural relaxation of confined carbon nanofibers and interlayer decoupling, with a potential secondary contribution from moderate p-doping imparted by residual oxygen functional groups. On the other hand, the almost zero variation in ID/IG (in both HCM-NaY and HCM) indicates minimal oxidation [68]. Elemental analysis by EDS and mapping corroborate the carbon coating of zeolite surfaces, with oxygen groups consistent with both partial oxidation and geometric confinement during synthesis. Figure 6b compares HCMs with commercial graphene oxide, multiwall carbon nanotubes, single-wall carbon nanotubes, and graphene. As shown in Figure 6b, the HCM Raman signal shows an increased D band compared with all commercial carbon allotropes, a shift in the 2D band, and a decrease in the intensity of the band, which implies an increase in disorder of the carbon structure, as previously observed, and a similarity to the Raman spectrum of graphene oxide (GO). Table 4 includes the intensity values of D, G, and the 2D band, and their ratios [36].

To evaluate the effect of acid on the samples, commercial graphene (Graphene Nanopowder 12nm flakes: AO-3, Graphene Supermarket, Ronkonkoma, NY, USA) was used as a control and processed with HF at the same time as HCM. The samples were analyzed by Raman microscopy before and after the acid treatment.

**Figure 7:**
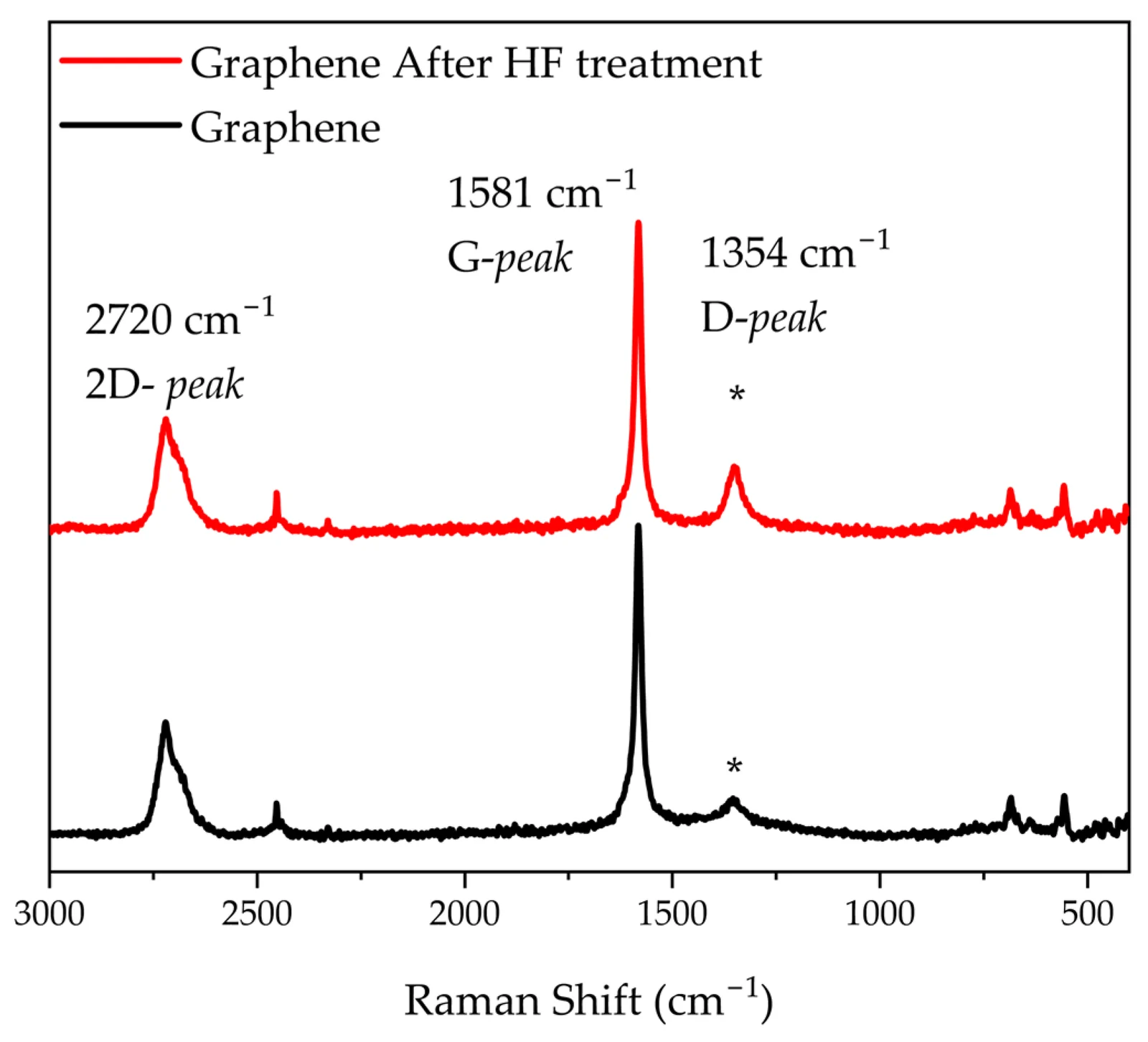
Graph of Raman microscopy analysis. Commercial graphene was used as a control to evaluate structural and functional group changes following hydrofluoric acid treatment. The black line corresponds to commercial graphene without any treatment. The red line corresponds to the same graphene processed under the same conditions and at the same time as the HCM−NaY sample, for the removal of zeolite NaY. The D-peak (marked with *) increased after the HF treatment, indicating increased disorder or defects.

Figure 7 shows commercial graphene before and after the HF treatment. Based on EDS mapping analysis, these defects are consistent with the incorporation of oxygen functional groups (C-Ox) bonded to carbon and entering the zeolite structure. The ID/IG ratio of commercial graphene (CG) is 0.13, and that of CG after acid treatment is 0.21. These values imply a small increase in oxidation and disorder after the HF treatment. Figure 7 shows the signal intensity of the D-peak (marked with an *) [68]. Comparing the ID/IG of HCM (0.74) with HCM-NaY (0.75), no variation is observed (Figure 6a), demonstrating a material resistant to HF and confirming that the shift in HCM is due to the zeolite removal from the structure, and not another type of chemical modification. In addition, the HCM I2D/IG ratio (0.06) is lower compared with HCM-NaY (0.19), which suggests higher oxidation rather than structural disorder (cf. Table 4).

### 2.6. Synthesis Temperature Selection

**Figure 8:**
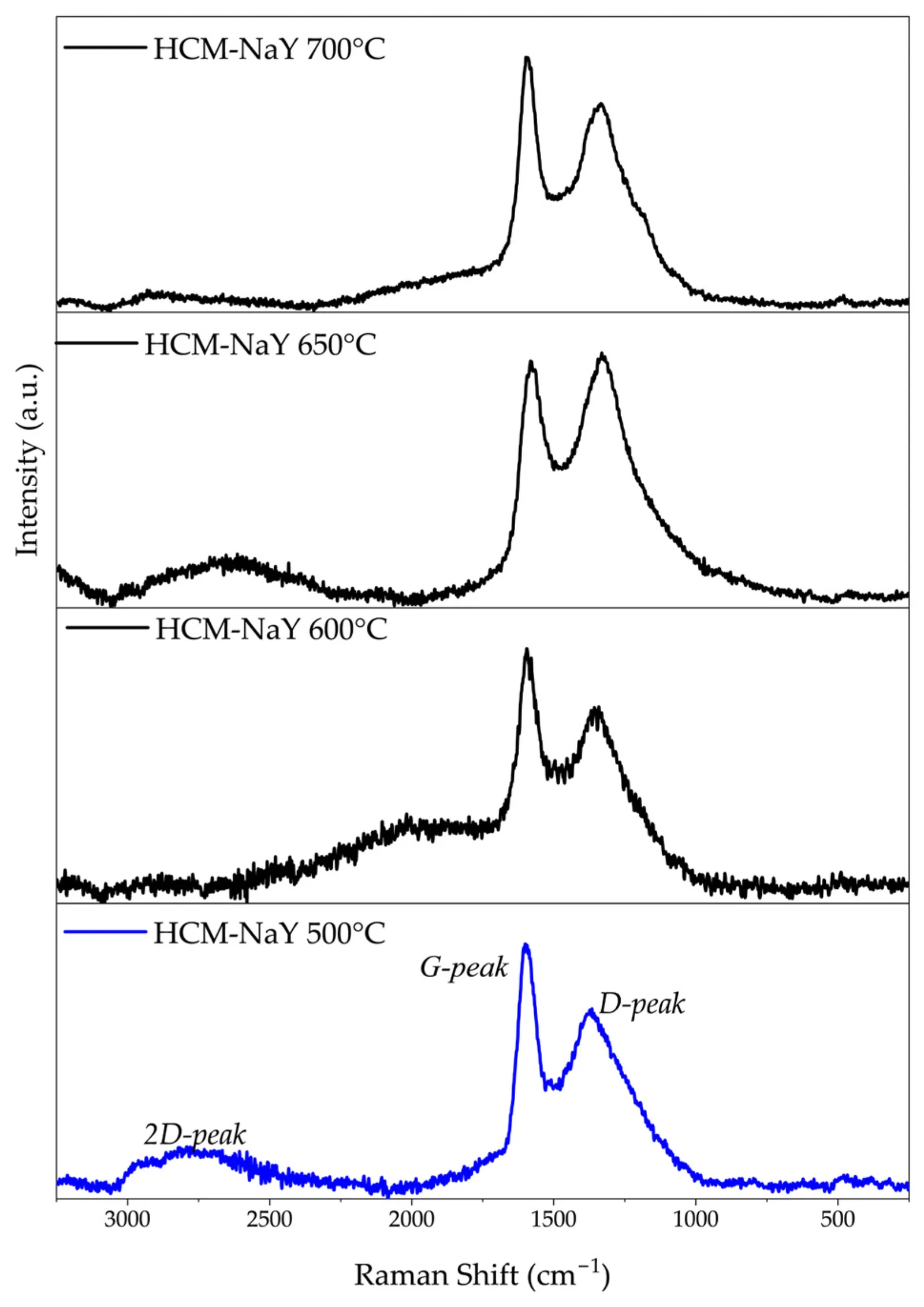
Graph of Raman microscopy analysis. The analysis was performed using a 532 nm laser at 10 mW. The blue line corresponds to the hybrid carbon material with NaY zeolite (HCM-NaY) synthesized at 500 °C. The black lines correspond to 600, 650, and 700 °C. The other physicochemical parameters were fixed (pressure, flux, carbon feedstock).

Raman spectroscopy was used to evaluate the working temperature of the HCM synthesis (Figure 8). The synthesis was performed at 500, 600, 650, and 700 °C, with all other physicochemical parameters held constant. The materials obtained were compared at the point after synthesis to determine how to continue to obtain pure HCM. The selection parameters were the ID/IG and I2D/IG ratios. Our criteria were to minimize ID/IG value while maximizing the I2D/IG ratio. Based on the obtained values (see Table 5), the best results were obtained at a temperature of 500 °C. The lower ID/IG ratio indicates a lower number of disorders in its structures, as provided by the C-Ox functional groups [68]. The 2D-peak corresponds to the overtone of the D-peak, and as shown in HCM-NaY 500 °C (wide and between 2500 and 3000 cm^−1^) represents a disordered nanocrystal [68]. Although the sample synthesized at 600 °C (HCM-NaY 600 °C) shows an I2D/IG ratio close to the I2D/IG ratio, it is lower than that of the 500 °C sample (HCM-NaY 500 °C). While the highest I2D/IG value corresponds to HCM-NaY 650 °C, its significantly larger ID/IG value implies a structure with higher defects and disorder.

### 2.7. BET Surface Area

The Brunauer–Emmett–Teller (BET) method uses nitrogen isotherms to determine the surface area of material. The isotherms are cycles of nitrogen adsorption–desorption which allow the determination of porosity and surface area.

**Figure 9:**
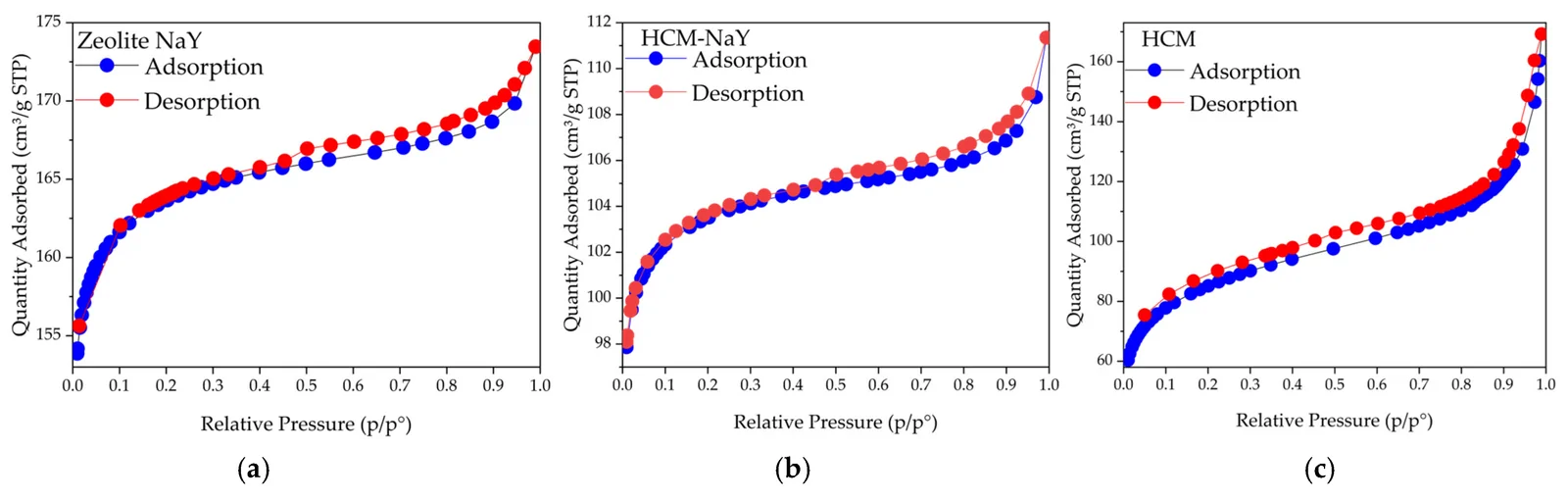
Isotherms of adsorption–desorption of (**a**) zeolite NaY (scaffold), (**b**) HCM-NaY, and (**c**) hybrid carbon material (HCM).

Figure 9 displays the isotherms for zeolite NaY (a), HCM-NaY (b), and hybrid carbon material (c). The observed isotherm in HCM is type II, which resembles non-porous, macroporous, or flat surfaces [69]. The plot is indicative of multiple layers and at pressures closer to saturation represents infinite adsorption. Further, the isotherms show hysteresis. All samples present type 3 hysteresis (H3 or type C). This results from a mixture of tapered or wedge-shaped pores with open ends [36,70]. This type of hysteresis is found in solids or particle agglomerates that present slit-shaped pores [70].

Both the hysteresis and the isotherm cycles provide characteristics of the material. For instance, the t-plot is used to calculate the pore size and the surface area is measured by the use of the BET method (Table 6). The surface area of the framework corresponds to 803 m^2^·g^−1^. The maximum of the area corresponds to the micropore (765 m^2^·g^−1^), which corresponds to an ordered structure similar to an ordered cage that includes channels [36]. A 3.5% decrease in surface area is observed after the HCM grows around the framework and penetrate the channels (HCM-NaY 775 m^2^·g^−1^). The micropores are reduced from 765 m^2^·g^−1^ to 607 m^2^·g^−1^, and the external surface increases (37 to 168 m^2^·g^−1^) [36]. This finding suggests that carbon fills the pores and that the HCM growth reproduces the framework structure. The difference between the framework surface area and zeolite with carbon materials (28 m^2^·g^−1^) is lower than the HCM only (296 m^2^·g^−1^), with the external surface area accounting for approximately 80% of the total (238 m^2^·g^−1^). These data suggest that carbon deposition occurs predominantly on the external surface rather than within the zeolite channels, resulting in macropore formation. This evidence is further supported by the HR-SEM (Figure 1c) and TEM (Figure 4) analyses. Based on the isotherm evidence, the resulting material corresponds to a mix of macropores (80%) and micropores (20%). The hysteresis data confirms that some internal micropores were blocked (difference in the desorption gas amount). These findings demonstrate that HCMs are promising candidates for applications demanding high interfacial surface area.

Taking the sum total of these data into account, we observe that the black powder obtained is composed of different carbon materials, such as graphene, graphene oxide, CNTs, and CNFs. This material grows both inside and around the framework. Singh and co-workers, in their research, conclude that this material can be considered a hybrid material [71], and consistent with Nishihara and co-workers [42], our XRD and BET results indicate that a type II material has been obtained [36].

## 3. Materials and Methods

The molecular sieves, sodium Y zeolite and Faujasite (zeolite, 100% purity, 334448), and hydrofluoric acid (48% *w*/*v* for analysis EMSURE^®^ ACS, ISO, Reag. Ph Eur, 1.00334) were purchased from Sigma Aldrich (St Louis, MO, USA). The annealed nickel foil, 0.025 mm thick (99% purity, metal basis, 012722RX), and acetone (>99.9% purity, A16P-4) were obtained from Fisher Scientific (Morris Plains, NJ, USA). They were used directly without any further purification.

### 3.1. Synthesis

The materials were obtained by the chemical vapor deposition method using a Barnstead Thermolyne 21100 Tube Furnace (Dubuque, IA, USA), model F21124. To start the synthesis, a Ni box (40 × 20 × 3 mm) filled with molecular sieves (zeolite NaY) was placed inside a quartz tube in the oven. The nickel:zeolite ratio was 4:3. The nickel box with zeolite was dried inside the tube furnace for 30 min at 500 °C using flowing Ar/H_2_ (90/10%) as the carrier and drying gas at 5.5 ± 1 kPa. Other growth temperatures (600, 650, and 700 °C) were evaluated to optimize the synthesis conditions to obtain quality HCM at the lowest working temperature (see Figure 8 and Table 5). The acetone feedstock enters the system through a flask reservoir of pure acetone at 48 kPa. The acetone is bubbled with Ar/H_2_ (90/10%) carrier gas, and the resulting vapor flows through the quartz tube, which contains the nickel box with zeolite. The flux (0.5 mL·min^−1^) was regulated with a vacuum pump, an Edwards18 pump (Edwards Vacuum, Haverhill, MA, USA) provided with a valve to a total pressure in the tube furnace of 46 ± 1 kPa for 60 min. The material obtained was detached from the zeolite by a three-step procedure consisting of acid digestion (3 h in a 48% *w*/*v* aqueous hydrofluoric acid), membrane filtration (0.22 µm polyvinylidene fluoride (PVDF), GVWP02500 Millipore, Sigma Aldrich), and aqueous washing (Milli-Q water, 18.00 MΩ·cm^−1^, own-produced with ARIES Filter Works, Camden, NJ, USA). The resulting material was dried in a hot plate (IKA^®^ C-MAG HS7, Staufen im Breisgau, Germany) at 40 °C for 12 h prior to characterization. This synthesis was repeated 60 times to verify that the results were consistent and reproducible [36].

### 3.2. Characterization

The resulting material was characterized by high-resolution scanning electron microscopy (HR-SEM) in a Thermo Scientific Phenom Pharos G2 Desktop FEG-SEM (Thermo Fisher Scientific, Waltham, MA, USA) equipped with a silicon drift detector (SDD) with an active area of 25 mm^2^, featuring an ultra-thin silicon nitride (SiNx) window that enables detection of elements ranging from boron (B) to californium (Cf). The system offers an energy resolution of ≤132 eV at Mn Kα and processes spectral data using a multi-channel analyzer with 2048 channels at a dispersion of 10 eV/channel, capable of handling a maximum input count rate of 300,000 counts per second (cps). Additional characterization was done by: transmission electron microscopy (TEM) in a Hitachi TEM system (Tokyo, Japan) HT7800 Ruli TEM; X-ray diffraction (XRD) in a Rigaku SmartLab III (Hokuto, Japan) equipped with a Cu-Kα (λ = 1.5417 Å) radiation source, operated with 40 kV of accelerating potential with a tube current of 44 mA; Raman spectroscopy in a DXR Raman Microscope from Thermo Scientific (Milwaukie, WI, USA); and BET surface area analysis in a TriStar II 3020 Micromeritics (Norcross, GA, USA) [36].

## 4. Conclusions

This research presents a hybrid nanomaterial obtained by chemical vapor deposition synthesis method using acetone vapor as carbon feedstock under low-pressure and low-temperature conditions. The characterization data indicates that a hybrid material composed of graphene, graphene oxide, carbon nanofibers, and carbon nanotubes results. The TEM images confirm the presence of CNFs and CNTs with different diameters, as observed by HR-SEM. The elemental composition obtained from EDS and mapping shows primarily carbon, oxygen, and trace amounts of nickel, aluminum, and fluorine, which do not interfere with the crystalline structure or vibrational modes. The crystalline size and d-spacing were obtained to explain the oxidation grade, the morphology of the solid particles, and the obtention of a hybrid carbon material. Raman spectroscopy confirms the growth of material around and within the zeolite structure and, based on vibrational modes, confirms that the structural disorder is mainly caused by the zeolite scaffold rather than oxidation by HF. BET analysis indicates that the material is a mixture of macroporous and microporous materials, with a low surface area, but indicates that the material occupies the entire surface of the zeolite. The combination of the CVD methodology followed by etching of the molecular scaffold provided a hybrid carbon structure with high purity (83.86 ± 0.43% carbon weight), a 35.12 ± 5.17% SRY, and heterogeneous morphologies. The resulting material has been applied as electrode material for energy storage in lithium-ion batteries (work in progress), and as a gas adsorbent for CO_2_. In addition, the material may have potential as a supercapacitor or for water filtration.

## Figures and Tables

**Table 1:** EDS results of the composition by weight percentage of zeolite-NaY, zeolite-HCM, and hybrid carbon material only. ND indicates not detected.

Material	C Weight %	O Weight %	Na Weight %	Al Weight %	Si Weight %
MS-NaY	ND	49.88 ± 0.01	8.56 ± 0.01	11.12 ± 0.01	30.26 ± 0.01
HCM-NaY	14.85 ± 0.02	47.94 ± 0.01	7.62 ± 0.01	8.25 ± 0.01	21.35 ± 0.01
HCM	85.26 ± 0.01	11.87 ± 0.01	ND	0.61 ± 0.01	ND

**Table 2:** Statistical information obtained from the plot of the size distribution. In total, 300 diameters were measured from the TEM images.

		N Total	Mean	Minimum	Maximum
Zeolite-HCM	CNTs	300	13.11 ± 3.6	4.30	26.00
	CNFs	300	53.82 ± 16.38	22.70	99.57
HCM	CNTs	300	7.21 ± 2.34	3.08	13.00
	CNFs	300	58.71 ± 12.92	36.89	99.00

**Table 4:** D, G, and 2D intensities, along with their ID/IG and I2D/IG ratios.

Material	ID	IG	I2D	ID/IG	I2D/IG
HCM-NaY	0.75	1.00	0.19	0.75	0.19
HCM	0.74	1.00	0.06	0.74	0.06
Commercial graphene	0.13	1.00	0.38	0.13	0.38
Commercial graphene, after HF	1.31	1.00	1.48	1.31	1.48
Graphene oxide	0.99	1.00	0.27	0.99	0.27
MWCNTs	0.18	1.00	0.57	0.18	0.57
SWCNTs	0.05	1.00	0.15	0.05	0.15

**Table 5:** Relative intensities and intensity ratios obtained from Raman spectroscopy at different temperatures. The ID, IG, and I2D values, along with the ID/IG and I2D/IG ratios, are included over a range of working temperatures from 500 to 700 °C.

Material	ID	IG	I2D	ID/IG	I2D/IG
HCM-NaY 500 °C	0.75	1.00	0.19	0.75	0.19
HCM-NaY 600 °C	0.77	1.00	0.10	0.77	0.10
HCM-NaY 650 °C	1.00	0.97	0.19	1.03	0.20
HCM-NaY 700 °C	0.82	1.00	0.04	0.82	0.04

**Table 6:** Information obtained from the BET plot and t-plot analysis [36].

Material	BET m^2^·g^−1^	Volume of Monolayer cm^3^·g^−1^	t-Plot External Surface Area m^2^·g^−1^	t-Plot Micropore Area m^2^·g^−1^	t-Plot Micropore Volume cm^3^·g^−1^
Zeolite NaY	803	185	37	765	0.35
HCM-NaY	775	178	168	607	0.27
HCM	296	68	238	58	0.0023

## Data Availability

The original contributions presented in this study are included in the article. Further inquiries can be directed to the corresponding author.
